# Characterization of *Pseudomonas aeruginosa* Bacteriophage L5 Which Requires Type IV Pili for Infection

**DOI:** 10.3389/fmicb.2022.907958

**Published:** 2022-07-01

**Authors:** Lan Yang, Tingting Zhang, Linlin Li, Chao Zheng, Demeng Tan, Nannan Wu, Mingyang Wang, Tongyu Zhu

**Affiliations:** ^1^Shanghai Institute of Phage, Shanghai Public Health Clinical Center, Fudan University, Shanghai, China; ^2^Key Laboratory of Infectious Immune and Antibody Engineering of Guizhou Province, School of Biology and Engineering, Guizhou Medical University, Guiyang, China; ^3^Department of Critical Care Medicine, Jiangbei District People’s Hospital, Chongqing, China; ^4^CreatiPhage Biotechnology Co., Ltd, Shanghai, China; ^5^Shanghai Medical College, Fudan University, Shanghai, China

**Keywords:** *Pseudomonas aeruginosa*, phage (bacteriophage), *Autographivirinae*, phage-host interaction, phage receptors

## Abstract

*Pseudomonas aeruginosa* is a common opportunistic human pathogen. With the emergence of multidrug-resistant (MDR) clinical infection of *P. aeruginosa*, phage therapy has received renewed attention in treating *P. aeruginosa* infections. Moreover, a detailed understanding of the host receptor of lytic phage is crucial for selecting proper phages for therapy. Here, we describe the characterization of the *P. aeruginosa* bacteriophage L5 with a double-stranded DNA genome of 42,925 bp. The genomic characteristics indicate that L5 is a lytic bacteriophage belonging to the subfamily *Autographivirinae.* In addition, the phage receptors for L5 were also identified as type IV pili, because the mutation of *pilZ*, which is involved in pili synthesis, resists phage infection, while the complementation of *pilZ* restored its phage sensitivity. This research reveals that L5 is a potential phage therapy candidate for the treatment of *P. aeruginosa* infection.

## Introduction

*Pseudomonas aeruginosa* is an important opportunistic human pathogen causing various acute and chronic infections, especially in cystic fibrosis (*CF*) patients, cancer patients, and immunocompromised individuals (Moradali et al., 2017). It is also a common pathogen that causes hospital-acquired infections, such as ventilator-associated pneumonia, urinary catheter-related infection, and surgical or transplantation infections (Moradali et al., 2017; Ibrahim et al., 2020).

In recent years, with the emergence of multidrug-resistant (MDR) *P. aeruginosa*, bacteriophages have been suggested as an alternative way to treat *P. aeruginosa* infections (Chegini et al., 2020; Yang et al., 2020). Many studies use phages to treat *P. aeruginosa* infections in animals (Forti et al., 2018; Cafora et al., 2019; Raz et al., 2019; Lin et al., 2021). In addition, phages were shown to effectively treat *P. aeruginosa* infection in patients with otitis, prosthetic knee, lung, acute kidney injury and burn wounds (Wright et al., 2009; Jennes et al., 2017; Aslam et al., 2019; Jault et al., 2019; Law et al., 2019; Ferry et al., 2021). However, one of the problems during bacteriophage therapy is phage resistance, which is often due to the mutation of receptors on the surface of the host bacterial. Therefore, it is critical to identify the receptor of new and diverse *P. aeruginosa* phages for their application to phage therapy in future.

Type IV pili (TFP) is a common bacterial surface structure in Gram-positive and Gram-negative bacteria. It confers many functions to bacteria, such as motility, adherence, DNA uptake, bacterial aggregation, and pathogenesis (Craig et al., 2004; Bahar et al., 2009; Denis et al., 2019). In addition, TFP plays a significant role in bacterial virulence and is a promising therapeutic target (Bahar et al., 2009; Dumenil, 2019). In *P. aeruginosa*, the biogenesis and function of type IV pili are controlled by over 40 genes. Some gene products are involved in biogenesis and mechanical function, whereas others play roles in transcriptional regulation and chemosensory pathways. Furthermore, the genes are expressed from unlinked gene clusters spread throughout the *P. aeruginosa* genome (Mattick, 2002; Burrows, 2012). Thus, identifying bacterial receptors for phages as type IV pili and characterizing the phage-host interaction mechanism is important in phage therapy and bacterial virulence.

To our knowledge, the majority of the *P. aeruginosa* phage receptors are the lipopolysaccharide (LPS; Jarrell and Kropinski, 1976, 1981; Temple et al., 1986; Yokota et al., 1994; Lam et al., 2011). While *P. aeruginosa* phage that targets type IV pili as receptors are not common (Heo et al., 2007; Bae and Cho, 2013). Therefore, to isolate phages that do not use the lipopolysaccharide (LPS) as a receptor, we use the *P. aeruginosa* strain PAO1r, an LPS O-antigen deficient mutant strain, as a host to isolate new phages.

In this study, we isolated a lytic bacteriophage, designated *P. aeruginosa* phage L5, from hospital sewage using the O-antigen deficient *P. aeruginosa* strain PAO1r as a host bacteria. *P. aeruginosa* phage L5 belongs to the subfamily *Autographivirinae.* In addition, we identified that phage L5 uses type IV pili as its receptor.

## Materials and Methods

### Bacterial Strains, Plasmids, Media, and Growth Conditions

Bacterial strains, bacteriophages, and plasmids used in this study are listed in Table 1. *Escherichia coli* and *P. aeruginosa* strains were routinely grown overnight at 37°C on Luria–Bertani (LB) solid medium or in LB broth with shaking at 220 rpm. The medium was supplemented with antibiotics at the following final concentrations: gentamicin (Gm), 10 μg/ml for *E. coli,* and 35 μg/ml for *P. aeruginosa*.

**Table 1:** Bacterial strain, phage and plasmids used in this study.

Strain, phage and plasmid	Relevant traits^a^	Source of reference
*Escherichia coli*		
SM10λpir	Conjugative donor strain	ZOMANBIO^b^
*P. aeruginosa* strain		
PAO1r	O-antigen deficient *P. aeruginosa*	(Shen et al., 2018)
PAO1rRL5	a phage L5-resistant mutant of PAO1r	This study
PAO1rΔ*pilZ* PAO1rΔ*pilZ:: pilZ*	PAO1r mutant with a deletion in the *pilZ* gene PAO1rΔ*pilZ* complemented with *pilZ*	This study This study
Phage		
L5	*P. aeruginosa* lytic phage	This study
Plasmids		
pEXG2	Allelic exchange vector, Gm^R^	(Rietsch et al., 2005)
pEXG2-*pilZ*	Deletion vector for deletion of *pil*Z	This study
pHB20TGm	Complementation vector, Gm^R^	a gift ^c^
pHB20TGm-*pil*Z	Complementation vector for complementation of *pil*Z	This study

a Gm^R^, gentamicin resistance.

b ZOMANBIO, Beijing Zoman Biotechnology Co., Ltd.

c a gift from Shuai Le (Army Medical University, Chongqing).

### Isolation and Purification of *Pseudomonas aeruginosa* Phage L5

*P. aeruginosa* phage L5 was isolated from hospital sewage using strain PAO1r as a host, based on a traditional double-layer agar method as described previously (Chen et al., 2019). Briefly, the sewage samples from Shanghai Public Health Clinical Center were centrifuged at 12,000 × g for 10 min and then filtered through a 0.22-μm pore size filter (Millex-GP USA). After that, 5 ml of the filtrate were mixed with 250 μl of log-phase PAO1r cells (OD_600_ = 0.6). After 6–8 h incubated at 37°C with shaking at 220 rpm, the mixture was centrifuged at 12,000 × g for 5 min. Then, the 300 μl supernatant was mixed with 500 μl host bacteria incubated at RT for 5 min, added to 5 ml of molten soft LB (0.5% agar), the mixtures were poured into LB plates and incubated at 37°C overnight. A single plaque was picked up and resuspended in SM buffer [5.8 g of NaCl, 2.0 g of MgSO_4_·7H_2_O, 50 ml of Tris–HCl (PH = 7.4), 5.0 ml of 2% gelatin]. The phage-containing SM buffer was filtered through a 0.22-μm pore size filter (Millex-GP USA) and subjected to serial 10-fold dilutions in sterile SM buffer to purify the phage. Phage purification was performed by double-layer agar plate method and repeated at least three times, and the purification phage was stored at 4°C in sterile SM buffer.

### Transmission Electron Microscopy

The morphology of the purified phage particles was observed using transmission electron microscopy (TEM). The phage particles were loaded on a carbon-coated copper grid to absorb for 15 min and negatively stained with 2% phosphotungstic acid (PTA, pH 7.0) for 2 min. Phage particles were observed using TEM (80 Kv, JEOL JEM-1200EXII, Japan Electronics and Optics Laboratory, Tokyo, Japan).

### The MOI of *Pseudomonas aeruginosa* Phage L5

The host strain PAO1r was cultured to log-phase (OD_600_ = 0.6) and adjusted to about 1 × 10^8^ CFU/ml. Phages were added according to the Multiplicity of Infection (MOI) of 1, 0.1, 0.01, 0.001, and 0.0001, respectively. The mixture was incubated for 3.5 h at 37°C with shaking at 220 rpm, centrifuged at 10,000 × g for 5 min at 4°C, and filtered through a 0.22-μm pore size filter. The titers were detected by the double-layer agar plate method. The experiment was repeated three times.

### The One-Step Growth Curve of *Pseudomonas aeruginosa* Phage L5

The host strain PAO1r was cultured in 5 ml LB broth until log-phase (OD_600_ = 0.6; ~1 × 10^8^ CFU/ml). The phage was added at a MOI = 0.01, and incubated at 37°C for 1 min without shaking. The mixture was then centrifuged at 10,000 × g for 3 min to remove free phage. Next, the precipitate was resuspended with 5 ml of fresh 37°C LB broth and cultivated at 37°C with shaking at 220 rpm. Samples were collected for 1 min, 5 min, 10 min, 20 min, 30 min, 60 min, 90 min, 120 min, and 150 min to determine the phage titer using the double-layer agar plate method. The above experiments were repeated three times.

### Host Range of *Pseudomonas aeruginosa* Phage L5

Spot test was used to determine the host range of *P. aeruginosa* phage L5 on 41 clinical *P. aeruginosa* strains (Supplementary Table S1). All the tested strains were cultured at log-phase; 300 μl of each tested strain was mixed with 5 ml molten soft 0.7% LB agar containing 300 μl of each test bacterial culture was overlaid on 1.5% LB agar plates. Subsequently, 10 μl (~10^9^ PFU/mL) of phage L5 was spotted on the soft agar. The result was observed after overnight incubation at 37°C.

### DNA Extraction and Bioinformatics Analysis of *Pseudomonas aeruginosa* Phage L5

The phage genomic DNA was extracted using the phenol-chloroform protocol as described previously (Chen et al., 2019). The genome of L5 was sequenced at the Beijing Novogene using IlluminaHiSeq 2,500 platform with 200 bp read length. Protein-encoding putative open reading frames (ORFs) were predicted using RAST, tRNAs were predicted using tRNAscan-SE 2.0. The virulence genes and antibiotic resistance genes were analyzed in the virulence factors database (VFDB; VFDB: database search mgc.ac.cn) and comprehensive antibiotic resistance database (CRAD),^1^ respectively. The phylogenetic tree of the phage large terminase subunit sequences was constructed using MEGA6 with 1,000 Bootstrap replications.

### Screening of Phage L5-Resistant Mutants

The process of isolating phage-resistant mutants has been described previously (Johnson and Lory, 1987). Briefly, the strain PAO1r culture was infected with phage L5 particles and plated on LB agar plates for 24 h at 37°C. Single-colony was isolated and purified at least three times. The double-layer agar plate method was used to verify the resistance of isolated mutants to phage L5.

### Twitching Motility Assay

Twitching motility assays were used as an indirect measurement of type IV pili function and described previously (McCutcheon et al., 2018). Briefly, a single bacterial colony was suspended in 100 μl LB broth and inoculated with a toothpick through a 3 mm thick LB agar layer (1% agar) to the bottom of the petri dish and incubated with 37°C for 72 h. Twitching motility zones between the agar and petri dish interface were visualized by gently removing the agar and staining each plate with 1% (w/v) crystal violet for 30 min followed by rinsing excess stain away with water. Stained twitching zone areas were measured using ImageJ software. Each strain was tested in biological triplicate, and the average twitching area was calculated from the three twitching zones.

### Bacteriophage *Pseudomonas aeruginosa* Phage L5 Adsorption Assay

Bacteriophage adsorption assay was performed as previously described with some modifications (Chibeu et al., 2009). Briefly, bacteria that grew overnight on the LB agar plate were resuspended with fresh LB broth and adjusted to OD_600_ = 0.6 (~1 × 10^8^ CFU/ml). Phage L5 was added to the bacterial suspension at an MOI of 0.01 and aliquoted in an equal volume to three microtubes. During the phage adsorption for 15 min at 37°C, each tube was centrifuged at13,000 × g for 5 min at the indicated time points, and the supernatants were immediately filtered through a 0.22-μm pore size filter (Millex-GP USA). The titer was immediately determined by the double-layer agar plate methods. The percentage adsorption was calculated as [(inter titer-the titer after adsorption)/inter titer] × 100. The final adsorption rate was obtained from three independent experiments.

### Construction of **Δ**pilZ *Pseudomonas aeruginosa* Strain PAO1r

The information of *pilZ* gene and the primers used in this study are listed in Supplementary Table S2; Table 2. In-frame deletion mutagenesis was used to construct *pilZ*-defective strain. Briefly, pilZ-up-F and pilZ-up-R were used to amplify the upstream of *pilZ*, pilZ-down-F and pilZ-down-R were used to amplify the downstream of *pilZ*. Secondly, pilZ-up-F and pilZ-down-R were used to produce the deletion fusion fragment by PCR, which has cutting sites of XhoI and XbaI. Thirdly, the XhoI/XbaI digested fragment was cloned into the XhoI/XbaI digested vector pEXG2, and transformed into SM10 λpir strain; after shaking at 37°C for 2 h, the bacterial was poured onto LB agar plate containing 15 μg/ml Gentamicin for 24 h at 37°C. Then, pilZ-up-F and pilZ-down-R were used to identify the vector pEXG2-*pilZ*. Parental strain PAO1r and the SM10 λpir strain with the vector pEXG2-*pilZ* were cultured at OD_600_ = 1.0, then the two strains were mixed and dropped on an LB agar plate for conjugation overnight. On the second day, scrape off the bacteria in fresh LB broth, shakes at 37°C for 1 ~ 2 h, and pour onto an LB agar plate containing 30 μg/ml gentamicin and 100 μg/ml arsenic trichloride. Next, purify clones on LB agar plates supplemented with 30 μg/ml gentamicin and 5% sucrose. Lastly, PCR and spot tests were used to identify the *pilZ*-deletion strain.

**Table 2:** Primers used in this study.

Primer	Sequence (5′-3′)	Function
pilZ-up-F	TTT*CTCGAG*CAAGGTCGTGTTGCTCGAAC (XhoI)	Amplification of *pilZ* upstream
pilZ-up-R	GCATGATCCTGTCTAGCGGCAGGTTCCTGCCAGTCGAATATCAGC	Amplification of *pilZ* upstream
pilZ-down-F	GACTGGCAGGAACCTGCCGCTAGACAGGATCATGCTGGTCGATTC	Amplification of *pilZ* downstream
pilZ-down-R	TTT*TCTAGA*CGACATCGCGCACGTATTCC (XbaI)	Amplification of *pilZ* downstream
pilZ-C-F	TTT*TCTAGT*ATGAGTTTGCCACCCAATC (XbaI)	Amplification of gene *pilZ*
PilZ-C-R	TTT*AAGCTT*TTACATCGTGTGGGTCG (HindIII)	Amplification of gene *pilZ*

### Complementation of *Pilz* Mutants

To complement *pilZ* in PAO1rΔ*pilZ*, the *pilZ* gene was amplified using primer pilZ-C-F and pilZ-C-R, and the PCR product was digested by HindIII/XbaI, and cloned into the HindIII/XbaI digested vector pHB20TGm. The product pHB20TGm-*pilZ* was electroporated into PAO1rΔ*pilZ*. Phage sensitivity of the transformants was analyzed by the efficiency of plaquing (EOP).

### Phage Plaquing Assay

The plaquing assay was determined by spotting phage on bacterial soft agar overlays. Briefly, 100 μl of overnight culture was mixed with 3 ml of soft 0.7% LB agar, overlaid onto 1.5% LB agar plates with or without antibiotics, and allowed to dry at room temperature for 30 min. Phage stocks were about 10^11^ PFU/mL on *p. aeruginosa* strain PAO1r and 10-fold serially diluted in SM to 10^1^ PFU/mL. 5 μl of each dilution was spotted onto the prepared plates and incubated for 18 h at 37°C. Each experiment was repeated in biological and technical triplicate.

## Results

### The Biological Characteristics of *Pseudomonas aeruginosa* Phage L5

**Figure 1:**
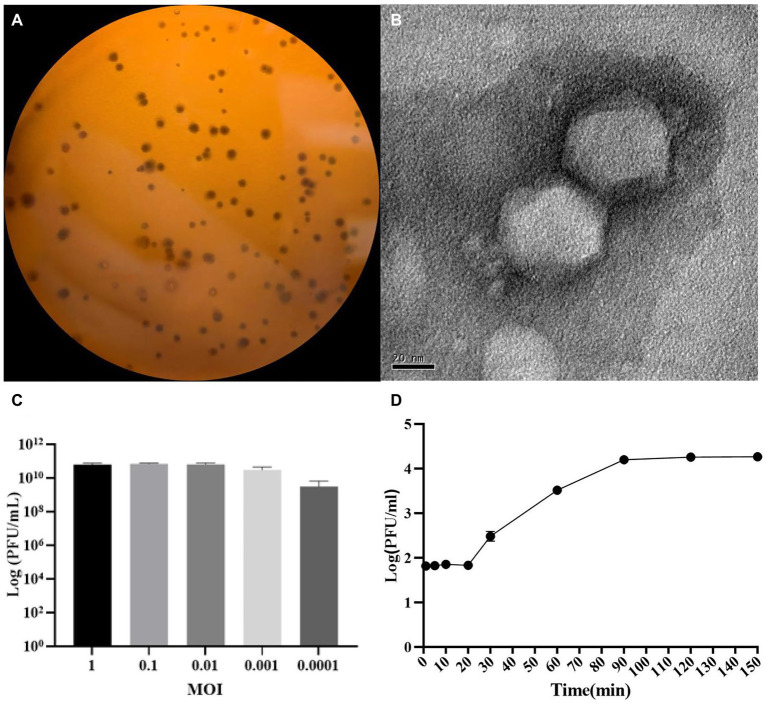
The morphological characteristics of *Pseudomonas aeruginosa* phage L5. **(A)** Plaque morphology of L5 on *P. aeruginosa* strain PAO1r; **(B)** Transmission electron micrograph image of L5. The scale bar represents 20 nm; **(C)** The multiplicity of infection (MOI) of L5; **(D)** The one-step growth curve of L5 on *P. aeruginosa* strain PAO1r. Error bars indicate standard deviation.

*P. aeruginosa* strain PAO1r was used as a host to isolate a lytic phage named *P. aeruginosa* phage L5. The results showed that the L5 could form clear plaques on LB double-layer agar plate (Figure 1A). The TEM showed that the L5 had an icosahedral head with a diameter of 55 nm and a very short noncontractile tail (Figure 1B); the morphology suggested that the L5 is a member of the *Podoviridae* family, order *Caudovirales*. The optimum MOI of the L5 was 0.01 (Figure 1C). The one-step growth curve of the L5 showed that its latent and burst period was approximately 20 min and 70 min, respectively (Figure 1D). In addition, the host range of the L5 was analyzed against 41 clinical isolated *P. aeruginosa* strains (Supplementary Table S1). The result showed that 13 *P. aeruginosa* strains could be lysed, including lost LPS O-antigen strain PAO1r.

### Genomic Analysis of *Pseudomonas aeruginosa* Phage L5

**Figure 2:**
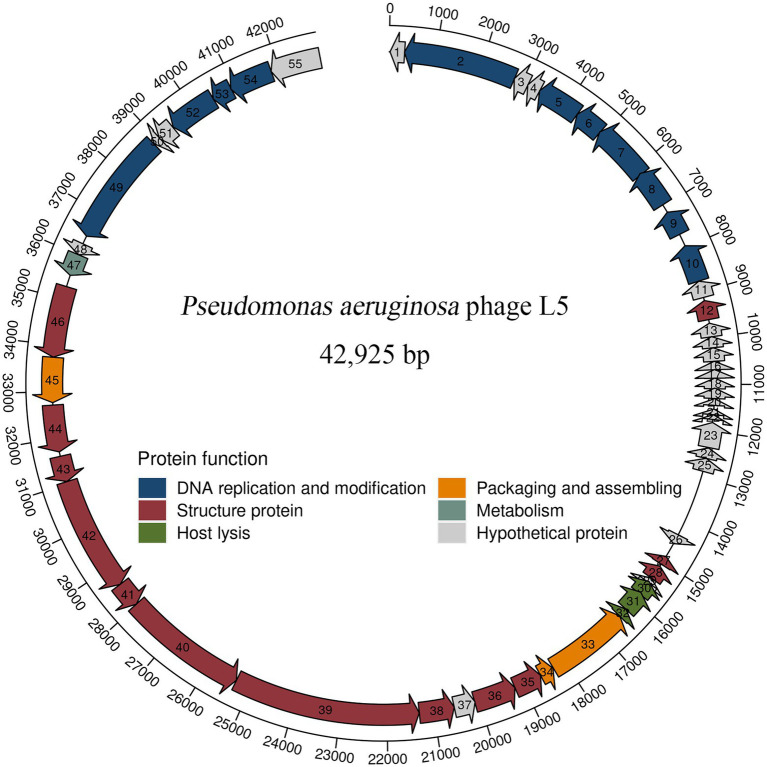
Genome organization of *P. aeruginosa* phage L5. Arrows represent predicted CDSs, with the direction of the arrow indicating the transcription direction. The different colors indicate different functional modules of gene products. The L5 genome can be divided into six functional modules.

The complete genome sequence of L5 and annotation information is deposited in GenBank (accession number OL754589). The genomic analysis revealed that the L5 has a linear double-stranded DNA comprising 42,925 bp with a G + C content of 48.1%. A total of 55 protein-coding genes were predicted in the L5 genome. In addition, blastP analysis revealed that 32 proteins had homologs to other proteins with known functions. Meanwhile, 23 proteins were assigned as hypothetical proteins, which is common among phage populations (Meira et al., 2016; Cai et al., 2021). The detailed annotation and organization of the L5 genome are illustrated in Figure 2.

L5 genome can be divided into six modules, including five functionally identified modules and one functionally unknown module. In five functionally identified modules, 11 ORFs encode DNA replication and modification proteins, 3 ORFs encode packaging and assembling proteins, 13 ORFs encode structure proteins, 1 ORF encodes metabolism protein, and 3 ORFs encode host lysis proteins (Figure 2). In addition, no tRNA genes, antibiotic genes, toxin genes, and lysogeny genes were predicted in the L5 genome. These results revealed that the L5 satisfies several recommended criteria for selecting a therapeutic phage (Philipson et al., 2018).

### Phylogenetic Analysis of *Pseudomonas aeruginosa* Phage L5

**Figure 3:**
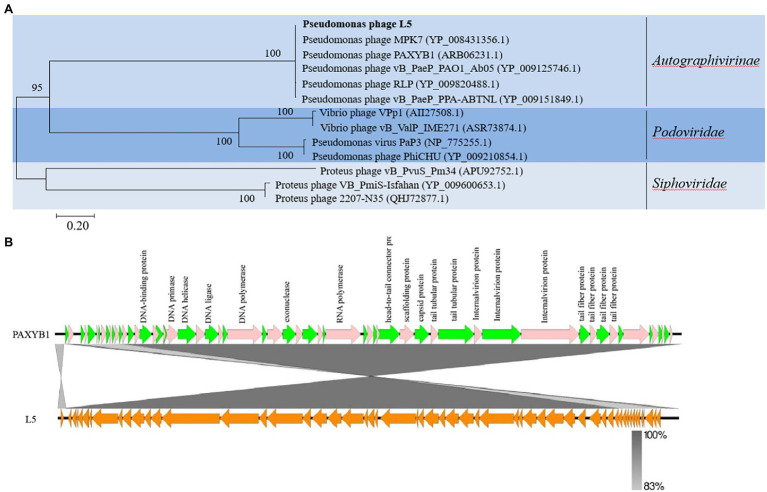
**(A)** Phylogenetic analysis of *P. aeruginosa* phage L5 and related phages. The terminase large subunits of selected phages were compared using the ClustalW program, and the phylogenetic tree was generated using the neighbor-joining method with 1,000 bootstrap replicates. **(B)** Comparison of the genome sequence of phage PAXYB1(top) with L5 (bottom). Predicted ORFs and the direction of transcription are indicated by block arrows. Conserved regions are shaded in gray. The color intensity corresponds to the sequence identity level (83 to 100%). Genomic comparisons were performed using BLASTn. Similarities with E values lower than 0.00001 are plotted. The figure was produced using Easyfig 2.2.5 (Sullivan et al., 2011).

To further explore the evolutionary position of the L5, the phylogenetic analysis of L5 and other related phages was analyzed using the neighbor-joining method. Phage terminase large subunits (TerL) is a relatively conserved protein and is mainly used as a phylogenetic marker in the comparative analysis of phage genomes (Feiss and Rao, 2012). Hence, the phylogenetic tree was constructed based on the TerL of these phages. In the phylogenetic tree of the TerL, L5 clustered together with the group including Pseudomonas phage MPK7 (YP_008431356.1), Pseudomonas phage PAXYB1 (ARB06231.1), Pseudomonas phage vB_PaeP_PAO1_Ab05 (YP_009125746.1), Pseudomonas phage RLP (YP_009820488.1) and Pseudomonas phage vB_PaeP_PPA-ABTNL (YP_009151849.1; Figure 3A). This result showed that L5 was the most closely related to the *Autographivirinae* subfamily. In addition, the sequences of L5 and PAXYB1 share 98.51% identity (Figure 3B). Therefore, combining the evolutionary analyses and some identical properties, including the morphology and linear genome, concluded that L5 is a new member of the subfamily *Autographivirinae*, the family *Podoviridae*, the order *Caudovirales*.

### Phage-Resistant Mutant Isolation and Sequencing

**Figure 4:**
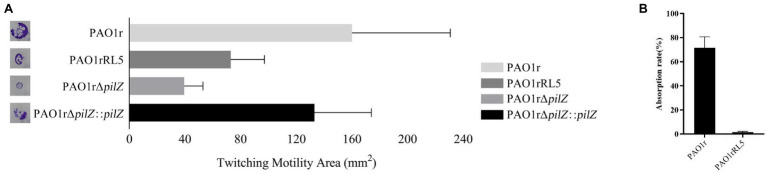
**(A)** Twitching motility zones by the wild-type sensitive strain PAO1r, the L5-resistant mutant strain PAO1rRL5, the knockout strain PAO1rΔ*pilZ*, and the complementation strain PAO1rΔ*pilZ*::*pilZ*. **(B)** Phage Adsorption Assay of *P. aeruginosa* phage L5 for the strain PAO1r and the strain PAO1rRL5. The phage adsorption percent was calculated as described in the MATERIALS and METHODS.

LPS is the most common receptor for bacteriophages. However, in this study, the L5 can infect strain PAO1r, an O-antigen deficient mutant strain. In this case, it revealed the receptor of L5 is not LPS. Therefore, to investigate the receptor of strain PAO1r to L5, a phage L5-resistant mutant strain PAO1rRL5 derived from the wild-type phage-sensitive strain PAO1r, was isolated and sequencing as described in Materials and methods. By comparative genomic analysis, we found that a type IV pili *pilZ* gene has a mutant compared to the genome of strain PAO1r. The detail information of *pilZ* gene is shown in Supplementary Table S2. The type IV pili is a well-characterized virulence factor in *P. aeruginosa*, involved in surface motility, biofilm formation, and adherence to mammalian cells and surfaces (Burrows, 2012). Furthermore, in *P. aeruginosa*, a pilZ domain-containing protein (PA2960) involved in twitching motility (Merighi et al., 2007). Thus, in order to determine whether the type IV pili *pilZ* gene can affect twitching motility, we tested the twitching motility assays using the wild-type sensitive strain PAO1r, the phage L5-resistant strain PAO1rRL5, the *pil*Z gene knockout strain PAO1rΔ*pilZ* and the *pil*Z gene complementation strain PAO1rΔ*pilZ*::*pilZ*. The result revealed that the phage L5-resistant strain PAO1rRL5 and the *pil*Z gene knockout strain PAO1rΔ*pilZ* displayed the twitching motility is reduced by 54 and 75% relative to the phage L5-resistant strain PAO1rRL5, compared to the *pil*Z gene complementation strain PAO1rΔ*pilZ*::*pilZ* restoring twitching motility to 83% of the phage L5-resistant strain PAO1rRL5. In addition, to perform the phage adsorption assay, about 71.43% of the L5 virions had adsorbed onto the strain PAO1r control cells. However, adsorption on the phage L5-resistant mutant strain PAO1rRL5 decreased to 1% (Figure 4). These results indicated that phage L5-resistant mutants prevent phage adsorption.

### *Pilz* is Responsible for *Pseudomonas aeruginosa* Phage L5 Infection

**Figure 5:**
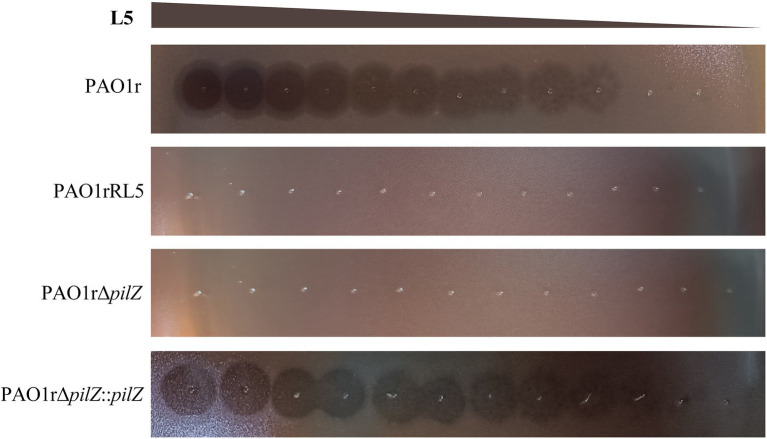
Phage Plaquing Assay of *P. aeruginosa* phage L5. The strain PAO1r and the strain PAO1rΔ*pilZ*::*pilZ* are susceptible to L5, while the strain PAO1rRL5 and strain PAO1rΔ*pilZ* are resistant to infection.

To further confirm the type IV pili *pilZ* gene is necessary for the infection of L5 to the host bacteria, we performed the phage plaque assay using the wild-type sensitive strain PAO1r, the phage L5-resistant strain PAO1rRL5, the *pil*Z gene knockout strain PAO1rΔ*pilZ* and the *pil*Z gene complementation strain PAO1rΔ*pilZ*::*pilZ*. The result showed that L5 loses sensitivity to the strain PAO1rRL5 and the strain PAO1rΔ*pilZ*. However, L5 restored the sensitivity to the complementation strain PAO1rΔ*pilZ*::*pilZ* (Figure 5). These results supported the type IV pili *pil*Z gene is required for L5 infection to the host strain PAO1r.

## Discussion

*Pseudomonas aeruginosa* is one of the most important bacterial pathogens with high mortality rates in patients diagnosed with cystic fibrosis, cancer, severe burns, and immunocompromised patients (Moradali et al., 2017). This bacterium can survive on water, different surfaces, and medical devices using influential binding factors such as flagella, pili, and biofilms (Moradali et al., 2017). The emergence of MDR *P. aeruginosa* strains led to phage therapy against *P. aeruginosa* has renewed interest (Chegini et al., 2020). Bacteriophage adsorption initiates the infection process (Rakhuba et al., 2010; Bertozzi Silva et al., 2016). Therefore, it is crucial in the infection process and plays an essential role in phage therapy against bacteria (Gordillo Altamirano and Barr, 2021). Thus, identifying phage receptors is vital and could expand phage therapy applications.

Type IV pili (T4P) are thin and flexible filaments found on the surface of a wide range of Gram-negative bacteria. T4P is involved in a broad range of functions, including twitching motility, adhesion, cell orientation, biofilm formation, pathogenicity, natural transformations, and bacteriophage infection (Craig et al., 2004; Persat et al., 2015; Tala et al., 2019). The type IV pili is a receptor for some *P. aeruginosa* phages has been identified before (Pemberton, 1973; Bae and Cho, 2013; McCutcheon et al., 2018); and in this study, we also identified the type IV pili *pil*Z gene is required for L5 infection. In addition, previous studies revealed that cyclic diguanylate (c-di-GMP) is a ubiquitous bacterial second messenger responsible for regulating cellular processes, including motility and biofilm formation. In *P. aeruginosa* genome encodes seven PilZ domain-containing proteins that have been shown to bind to c-di-GMP and an eighth PilZ domain protein that lacks c-di-GMP binding.

In this study, we found that the *pilZ* gene mutant led to a lower twitching motility zone. This means that if the *pil*Z gene has not mutant, leading to cell lysis when L5 infects the cells of the *P. aeruginosa* strain PAO1r. Meanwhile, the *pil*Z gene mutant leads to reduced motility. Therefore, the application of L5 may prove to be an effective therapy for *P. aeruginosa* infection.

In conclusion, we isolated a virulent bacteriophage L5. Genomic sequencing and analysis showed L5 belongs to the subfamily *Autographivirinae*, the family *Podoviridae,* and the order *Caudovirales*. Further, we identified the type IV pili as a receptor for phage L5 using genetic approaches.

## Data Availability Statement

The datasets presented in this study can be found in online repositories. The names of the repository/repositories and accession number(s) can be found at: https://www.ncbi.nlm.nih.gov/genbank/, OL754589.

## Author Contributions

ToZ and MW conceived and designed the experiments. LY and TiZ carried out the experiments and wrote the manuscript. NW, CZ, DT, and LL analyzed the data. MW and ToZ revised the manuscript. All authors contributed to the article and approved the submitted version.

## Funding

This research was supported by the Natural Science Foundation of Chongqing (Grant No. cstc2019jcyj-msxmX0794), the National Natural Science Foundation of China (Grant No. 82070772) and the Shanghai Public Health Clinical Center (Grant No. KY-GW-2021-27).

## Conflict of Interest

NW was employed by the company CreatiPhage Biotechnology Co., Ltd.

The remaining authors declare that the research was conducted in the absence of any commercial or financial relationships that could be construed as a potential conflict of interest.

## Publisher’s Note

All claims expressed in this article are solely those of the authors and do not necessarily represent those of their affiliated organizations, or those of the publisher, the editors and the reviewers. Any product that may be evaluated in this article, or claim that may be made by its manufacturer, is not guaranteed or endorsed by the publisher.

## Footnotes

^1^ https://omictools.com/card-tool
