# Supplementary material for: Characterization of Pseudomonas aeruginosa Bacteriophage L5 Which Requires Type IV Pili for Infection
Source: Front Microbiol. 2022 Jul 1;13:907958. doi: 10.3389/fmicb.2022.907958 (PMC9284122; doi:10.3389/fmicb.2022.907958)
Supplement: Supplementary file 1 [file Table_1.docx]

**Table S1** Host range of *Pseudomonas aeruginosa* phage L5

| Bacterial Strain | Isolated From | Resistance | Efficiency of Plating* | |
| --- | --- | --- | --- | --- |
| 2015364 | Secretions | carbapenem antibiotics | | - |
| 2015452 | Secretions | carbapenem antibiotics | | - |
| 2015647 | Secretions | carbapenem antibiotics | | **+** |
| 2015894 | Urine | carbapenem antibiotics | | **+** |
| 2015986 | Secretions | carbapenem antibiotics | | - |
| 2016218 | Secretions | carbapenem antibiotics | | - |
| 2016299 | Secretions | carbapenem antibiotics | | - |
| 2016657 | Secretions | carbapenem antibiotics | | **+** |
| 2016841 | Secretions | carbapenem antibiotics | | - |
| 2017911 | Secretions | carbapenem antibiotics | | - |
| 2018051 | Catheter | carbapenem antibiotics | | - |
| 2018052 | Secretions | carbapenem antibiotics | | - |
| 2018053 | Puncture fluid | carbapenem antibiotics | | **+** |
| 2018054 | Urine | carbapenem antibiotics | | - |
| 2018181 | Urine | carbapenem antibiotics | | **+** |
| 2018266 | Secretions | carbapenem antibiotics | | - |
| 2018318 | Urine | carbapenem antibiotics | | - |
| 2018321 | Secretions | carbapenem antibiotics | | - |
| 2018401 | Secretions | carbapenem antibiotics | | - |
| 2018399 | Catheter | carbapenem antibiotics | | - |
| 2033300 | Secretions | carbapenem antibiotics | | **+** |
| 2019144 | Secretions | carbapenem antibiotics | | **+** |
| 2019254 | Secretions | carbapenem antibiotics | | - |
| 2019688 | Secretions | carbapenem antibiotics | | **+** |
| 2019826 | Secretions | carbapenem antibiotics | | - |
| 2019925 | Secretions | carbapenem antibiotics | | - |
| 2020263 | Secretions | carbapenem antibiotics | | - |
| 2020392 | Secretions | carbapenem antibiotics | | - |
| 2020779 | Secretions | carbapenem antibiotics | | - |
| 2020804 | Secretions | carbapenem antibiotics | | - |
| 2021313 | Secretions | carbapenem antibiotics | | - |
| 2021354 | Secretions | carbapenem antibiotics | | - |
| 2021579 | Secretions | carbapenem antibiotics | | **+** |
| 2022346 | Secretions | carbapenem antibiotics | | - |
| 2022716 | Secretions | carbapenem antibiotics | | **+** |
| 2022717 | Urine | carbapenem antibiotics | | - |
| 2022718 | Urine | carbapenem antibiotics | | - |
| 2022923 | Urine | carbapenem antibiotics | | - |
| 2023046 | Secretions | carbapenem antibiotics | | **+** |
| 2023045 | Urine | carbapenem antibiotics | | **+** |
| H3 | Secretions | carbapenem antibiotics | | - |

*: (+) have plaques and (−) no plaques after infection with phage.
