# Supplementary material for: Characterization of Pseudomonas aeruginosa Bacteriophage L5 Which Requires Type IV Pili for Infection
Source: Front Microbiol. 2022 Jul 1;13:907958. doi: 10.3389/fmicb.2022.907958 (PMC9284122; doi:10.3389/fmicb.2022.907958)
Supplement: Supplementary file 2 [file Table_2.docx]

**TABLE S2** The information of *pilZ* gene in this study.

| **Name** | **Organism** | **Strain** | **Nucleotide Sequence** | **Protein sequence**  **(encode pilZ domain-containing protein)** |
| --- | --- | --- | --- | --- |
| *pilZ* gene | [Pseudomonas aeruginosa PAO1](https://www.ncbi.nlm.nih.gov/Taxonomy/Browser/wwwtax.cgi?mode=Info&id=208964)r | [PAO1](https://www.ncbi.nlm.nih.gov/Taxonomy/Browser/wwwtax.cgi?mode=Info&id=208964)r | ATGAGTTTGCCACCCAATCTGGGGCCGCGTAACGGCATCCTGTCCTTGACCATCAAGGACAAGTCCGTGCTGTACGCCGCCTACATGCCGTTCATCAGGAACGGCGGGCTGTTCATTCCCACCAACAAGAACTACAAGCTCGGCGACGAAGTCTTCATGCTGCTCAACCTGATGGAAGAGCCGGAGAAGATCCCGGTGGCCGGCAAGGTCGTCTGGATCACCCCGAAGGGCGCCCAGGGCAACCGTGCGGCTGGCATCGGCGTGCAGTTCAACGACGGTGACAACACCGCCCGCAACAAGATCGAAACCTACCTGGCCGGGGCGCTGAAGTCGGACCGGCCGACCCACACGATGTAA | MSLPPNLGPRNGILSLTIKDKSVLYAAYMPFIRNGGLFIPTNKNYKLGDEVFMLLNLMEEPEKIPVAGKVVWITPKGAQGNRAAGIGVQFNDGDNTARNKIETYLAGALKSDRPTHTM |
